# Physical activity, physical self-perception and depression symptoms in patients with major depressive disorder: a mediation analysis

**DOI:** 10.1007/s00406-021-01299-z

**Published:** 2021-07-19

**Authors:** Esra Görgülü, Miriam Bieber, Tobias Engeroff, Kirsten Zabel, Semra Etyemez, David Prvulovic, Andreas Reif, Viola Oertel

**Affiliations:** 1 Institute of Occupational, Social and Environmental Medicine, University Hospital of Frankfurt, Frankfurt am Main, Germany; 2 Institute of Occupational Medicine, Social Medicine and Environmental Medicine, Goethe-University Frankfurt am Main, Frankfurt am Main, Germany; 3 Department of Psychiatry and Behavioral Sciences, The Johns Hopkins University School of Medicine, Baltimore, MD USA

**Keywords:** Physical activity, MVPA, Physical self-perception, Self-esteem, Major depressive disorder

## Abstract

Physical inactivity is discussed as one of the most detrimental influences for lifestyle-related medical complications such as obesity, heart disease, hypertension, diabetes and premature mortality in in- and outpatients with major depressive disorder (MDD). In contrast, intervention studies indicate that moderate-to-vigorous-intensity physical activity (MVPA) might reduce complications and depression symptoms itself. Self-reported data on depression [Beck-Depression-Inventory-II (BDI-II)], general habitual well-being (FAHW), self-esteem and physical self-perception (FAHW, MSWS) were administrated in a cross-sectional study with 76 in- and outpatients with MDD. MVPA was documented using ActiGraph wGT3X + ® accelerometers and fitness was measured using cardiopulmonary exercise testing (CPET). Subgroups were built according to activity level (low PA defined as MVPA < 30 min/day, moderate PA defined as MVPA 30–45 min/day, high PA defined as MVPA > 45 min/day). Statistical analysis was performed using a Mann–Whitney *U* and Kruskal–Wallis test, Spearman correlation and mediation analysis. BDI-II scores and MVPA values of in- and outpatients were comparable, but fitness differed between the two groups. Analysis of the outpatient group showed a negative correlation between BDI-II and MVPA. No association of inpatient MVPA and psychopathology was found. General habitual well-being and self-esteem mediated the relationship between outpatient MVPA and BDI-II. The level of depression determined by the BDI-II score was significantly higher in the outpatient low- and moderate PA subgroups compared to outpatients with high PA. Fitness showed no association to depression symptoms or well-being. To ameliorate depressive symptoms of MDD outpatients, intervention strategies should promote habitual MVPA and exercise exceeding the duration recommended for general health (≥ 30 min/day). Further studies need to investigate sufficient MVPA strategies to impact MDD symptoms in inpatient settings. Exercise effects seem to be driven by changes of well-being rather than increased physical fitness.

## Introduction

Major depressive disorder (MDD) is associated with an increased risk of coronary heart disease and a significantly higher incidence of cardiovascular disease-related death [1]. For patients with depression, physical inactivity is discussed to be one of the most detrimental influences on cardiovascular health [2]. Previous studies indicated that people suffering from MDD are not only less physically active but also engage in higher levels of sedentary behaviour compared to matched controls [3].

Complementing evidence-based treatments of depression, such as psychotherapy and pharmacotherapy, a potential antidepressant effect of physical activity is discussed intensively [4] and bears the benefit of a low side effect profile.

Recent data show that about 50% of patients treated in psychiatric and/or primary care facilities discontinue antidepressant therapy prematurely [5]. The adherence to antidepressants is associated with concerns about side effects that may occur [6]. Adding to problems such as underdiagnosis or inadequate treatment approaches, around 30% of all patients fulfil criteria of treatment-resistant depression (TRD), i.e. lack of response to two courses of adequate treatment [7, 8]. This, along with the prevalence of depression overall [9] and the treatment gap for patients with mild and moderate depression [10], is one of the main reasons for the increasing importance attached to non-pharmacological treatment options from a public health perspective [11].

One potential target for treatment is the low level of physical activity linked to a higher risk of developing depression [12].

Theories implicate that interventions should focus on increasing the level of physical activity [13]. Furthermore, intervention studies indicate that more time spent with moderate-to-vigorous-intensity physical activity (MVPA) or exercise could lead to further improvement of depression symptoms, respectively [14, 15].

The World Health Organization (WHO; [16]) recommends adults (age 18–64 years) to engage in at least 150 min of moderate-intensity aerobic physical activity or at least 75 min of vigorous-intensity aerobic physical activity within 1 week in bouts of at least 10 min duration. Despite the potential positive effect of physical activity in MDD, comparably specific and clear recommendations are still lacking.

Although research has already shown that physical activity has an effect on depressive symptoms, the mechanisms involved are not yet sufficiently understood. In this respect, accelerometry might be a valuable source of additional information on patients with depression, especially since there is a lack of information on patients treated in outpatient—compared to inpatient settings.

The German psychiatric health care system is divided into inpatient care (the hospital sector) and outpatient care (medical care in hospital or in a psychiatric facility that does not involve an overnight stay). Differences between these two settings in terms of physical activity could help researchers and clinicians to identify specific environmental factors (for example, available facilities and differences in health policies, etc.) that should be taken into account.

A comparative meta-analysis in schizophrenic patients [17] showed already some interesting differences between levels of moderate and intense PA depending on the setting. A higher level of light physical activity was observed in community and outpatient settings than in inpatient settings. Another study by Vancampfort et al. [18] summarised data on sedentary behaviour and physical activity levels in people with schizophrenia, bipolar disorder and MDD in a global systematic review and meta-analysis. The results showed inpatients to be more active than outpatients and those living in the community. Against the prevailing background of low physical activity levels in patients with MDD, it is important to investigate these differences in this population more closely.

Brehm et al. [19] used a retrospective data analysis to investigate the actual extent of the use of exercise therapy in psychiatric inpatients in Germany. They were able to show that only 23% of these patients participated in exercise therapy with a mean exercise duration of 36 min/week. The study hereby underlined that the healthcare situation regarding exercise therapy is insufficient.

Furthermore, algorithms are needed to differentiate behavioural patterns such as habitual activities or structured exercises behind the positive effects of physical activity on depressive symptoms [20].

In addition to physical activity, increased cardiorespiratory fitness (CRF) has also an antidepressant effect [21]. To assess underlying physiological reactions, cardiopulmonary exercise tests (CPET) have become established as a safe method with accurate results [23]. Studies have already been able to show that low maximal oxygen uptake (*V*O_2max_) is associated with higher severity of depressive symptoms, or respectively, that an improvement in *V*O_2max_ predicts a greater reduction in depression severity. Future research should thus address the potential mediating influence of physical fitness on the link between PA and depression symptoms.

Studies conducted with healthy subjects have already shown that exercise has an effect on global self-esteem [22], self-efficacy and well-being improvement overall [22, 23]. In line with these results, Lubans et al. [24] have developed a conceptual model explaining the psychosocial and biological mechanisms for the effect of physical activity on cognitive and mental health in young people. The majority of these studies were able to show that improvements in physical self-perception and increased self-esteem are strongly connected to enhanced cognitive and mental health [25, 26]. Based on this data, another mechanism for PA effects could be based on changes in subjective well-being rather than in improvements in cardiorespiratory function or other parameters of physical fitness.

In a cross-sectional study with 67 schizophrenic patients, it was shown that high perceived sport competence and physical fitness (HPSCPF) are important determinants of participation in physical activity [27].

Patients with schizophrenia with HPSCPF performed more sports activities and were more physically active in their leisure time compared to patients with low perceived sports competence and perceived physical fitness (LPSCPF).

In addition, first studies have shown that physical activity can positively influence depressive symptoms by promoting self-esteem [23, 28]. However, studies that have examined the relationship between physical activity and self-esteem or general well-being in patients with mental illness, and especially those with MDD, remain scarce.

Regarding this background, the aim of this study was to analyse the influence of habitual PA and physical fitness on the severity of symptoms and well-being of inpatients and outpatients with MDD. For this purpose, we investigated associations between aerobic capacity (*V*O_2max_), habitual physical activity and subjective mediators such as self-esteem, physical self-perception and general well-being. Furthermore, we investigated whether meeting physical activity (PA) guidelines is sufficient, or if benefits on depressive symptoms can be derived from even greater volumes of PA. More precisely, we hypothesised that, (1) there is an inverse association between depressive symptoms and moderate-to-vigorous physical activity (MVPA). (2) Self-esteem, especially physical self-perception and general habitual well-being mediate the relationship between depressive symptoms and MVPA. (3) Depression symptoms decrease depending on activity level.

## Materials and methods

### Procedure

The data presented here are part of a prospective, controlled monocentric study on exercise and physical activity at the Department of Psychiatry, Psychosomatic Medicine and Psychotherapy, Goethe-University Frankfurt, Germany. All participants were given a description of the study and provided written informed consent before inclusion. Experimental procedures in this study were approved by the Ethics Committee (Registration-number 143/17, 15.12.17) of the University Hospital of the Goethe-University Frankfurt, Germany.

The study presented here is based on cross-sectional data collected in a baseline measurement of this prospective controlled monocentric study on exercise and physical activity.

Patients enrolled in the study were invited for a testing session following a medical check-up by a study physician. On the testing day, psychopathological assessments were conducted by psychologists blinded to the diagnosis and to any other test results. Furthermore, a physical fitness assessment was conducted using bicycle ergometry. Finally, the participants were given an ActiGraph wGT3X + ® triaxial accelerometer to record physical activity for 1 week.

### Participants

Participants were recruited in Frankfurt am Main (Germany) between August 2014 and March 2018. Inpatients were recruited from the Department of Psychiatry, Goethe-University, Frankfurt. Outpatients were addressed via articles in the local press and advertisements.

In total, 76 patients fulfilled the inclusion criteria and were enrolled in this study. Inclusion criteria for participants were an age between 18 and 65 [mean age (*M*) 39.90 ± 14.32], being fluent in German language and able to fill out the questionnaires. All participants were diagnosed with MDD according to DSM-IV criteria (SCID-I and SCID-II; German version: [29]) [mean age (*M*) = 47.61, SD = 12.22, 57 females, 19 males] by experienced clinicians. Additionally, the patients were screened using the BDI-II [30] (BDI-II at baseline for inpatients: 24 (min. 1, max. 48), for outpatients: 21 (min. 0, max. 39), BDI cut-off ≥ 10 indicating mild-to-moderate depression. The average duration of illness was (*M*) = 11.87 (SD = 11.55) years.

69 out of 76 patients were medicated with antidepressant medication (for detailed information about medication see Table 1). The amitriptyline equivalents for each MDD patient were computed as described by Ali [31] and the chlorpromazine equivalents using the formula by Woods [32]. To exclude biases by changed medication we ensured that all enrolled participants got stable medication as well as psychotherapy the last 4 weeks before testing.

**Table 1:** Sociodemographic and clinical characteristics of study participants by subgroup

	*M* (SD)			Sig. (*p*)
	Total (*N* = 76)	Inpatients (*N* = 31)	Outpatients (*N* = 45)	
*Sociodemographic characteristics*				
Age	47.61 (12.22)	41.58 (13.81)	51.76 (9.03)	< 0.001
Gender (female:male)	57:19	20:11	37:8	< 0.001
Years of education	13.13 (3.66)	13.55 (3.93)	12.85 (3.48)	0.459
*Psychiatric and physiological characteristics*				
Duration of illness (years)	11.87 (11.55)	9.79 (10.46)	12.84 (3.49)	0.698
Number of depressive episodes	4.94 (5.55)	3.04 (1.45)	5.79 (6.33)	0.129
AMI equivalent	124.17 (124.85)	84.30 (109.57)	182.04 (124.56)	0.575
CPZ equivalent	39.94 (85.85)	68.33 (107.84)	20.39 (58.16)	< 0.001
BMI	27.50 (5.48)	26.07 (5.30)	28.46 (5.45)	0.991
*V*O_2max_ ml/(min kg)	26.00 (7.39)^(*N*=24)^	23.55 (5.69)^(*N*=7)^	31.93 (8.05)^(*N*=17)^	0.413
Watt/kg	1.06 (0.65)^(*N*=64)^	1.80 (0.62)^(*N*=26)^	1.46 (0.65)^(*N*=38)^	0.993

Sample sizes vary as not all patients participated in all investigations completely

*M *mean, *SD* standard deviation, *AMI* amitriptyline, *CPZ* chlorpromazine

In addition, all patients underwent a medical check-up by a study physician to exclude medical contraindications for participation. Exclusion criteria for participants were meeting criteria for any comorbid Axis-I or II disorders or suffering from acute psychosis (including depressive disorders with psychotic features), significant medical disorders that would be a contraindication to maximal exercise testing, inability to give informed consent, and drug dependence or abuse (for minimum 1 year preceding the study).

64 of the 76 patients took part in bicycle ergometry to assess their physical fitness. Only 24 participants accepted the additional measurement of *V*O_2max_, while the remaining 40 patients did not tolerate the airtight face mask or rejected it from the outset.

### Physical fitness assessment

The participants performed a standardized incremental maximal exercise test on a bicycle ergometer (Model Ergometer GE Healthcare eBike L).

The initial stage (25 or 50 W) was chosen on the basis of gender, age, body weight, and training history with the expectation to reach a total exercise duration of at least 10 min.

Based on estimated fitness, we applied either a protocol of initially 25 W and increasing by 15 W every 3 min or 50 W and increasing by 25 W every 3 min at approximately 55–65 revolutions per minute (rpm) until exhaustion occurred. This was followed by active recovery, i.e., continuing without resistance for 3 min until the baseline heart rate was reached. The blood pressure was measured at rest and every 3 min during exercise test, while the heart rate was registered continuously using a 12-lead ECG (Kiss, GE Healthcare, Munich, Germany).

The maximal achieved workload was assessed and adjusted for weight (*p*(max)/kg). To assess spiroergometric parameters (*V*O_2_, CO_2_ production), the participants were wearing a face mask, which was connected to a spiroergometric system (oxycon Mobile, Viasys Healthcare GmbH, Wuerzburg, Germany). *V*O_2peak_ (in ml *V*O_2_ per minute per kg body weight) was operationalized as the highest oxygen uptake at the end of the exercise test.

The subjective exhaustion level was determined by the Borg Scale [33], while the affective changes were monitored applying the Feeling Scale [34] initially and then every 3 min towards the end of each stage until the recovery period was complete.

### Instruments

#### Psychopathology

All instruments have been used in German language. To assess MDD related psychopathology, the Beck Depression Inventory Revision (BDI-II, cut-off ≥ 10 indicating mild-to-moderate depression) [30] was used.

#### Self-esteem and physical self-perception

Furthermore, self-esteem was retrieved using the Multidimensional Self-Value Scale (MSWS) [35] (subscale total self-esteem). This questionnaire captures various manifestations of self-value. Based on the multi-facetted model of self-esteem according to Shavelson et al. [36], 6 different self-esteem facets are determined by 32 questions. For this study, the total self-esteem and the physical self-perception were used.

#### General well-being

Overall well-being, satisfaction with the current state of the body and physical ailments and pain were recorded using the FAHW [37] questionnaire. The FAHW questionnaire detects the general, i.e. physical, mental and social well-being in a positive and negative sense. The questionnaire consists of six scales. The items are each answered on a five-point Likert scale. For this study, the overall well-being score was considered.

#### Accelerometry: measurement of physical activity

ActiGraph wGT3X + ® triaxial accelerometers were used to objectively measure physical activity during daily routine.

The participants were instructed to wear the accelerometer, attached to the non-dominant waist by an adjustable belt, during all waking hours for at least 4 consecutive days, except during water-based activities. The criterion to validate daily PA data was a minimum recording of 8 h/day [38]. The sampling epoch has been set at 10 s [39]. Time per day spent in moderate physical activity (MPA; 1952–5723 counts per minute, > 3 MET) and vigorous physical activity (VPA; > 5274 counts per minute, > 6 MET) has been recorded based on the raw accelerometer counts with cut-off values derived from Freedson et al. [40]. For data management, the accelerometer ActiLife v.6.10.1® software was used.

### Statistical analysis

Data were analyzed for normal distribution using the Shapiro–Wilk test. Given the non-normally distributed data further analysis was performed using a Mann–Whitney *U* test, Spearman correlation and mediation analysis. *p *values   0.05 were considered statistically significant.

Medication (CPZ and AMI equivalents), age and gender were identified as possible confounding factors, and their influence on the results was investigated using regression analysis, correlation analysis and the Kruskal–Wallis test.

First, a simple linear regression method was used to assess the relationship between BDI-II score and MVPA.

Subsequently, a multiple linear regression model was constructed including all variables (age and gender as possible confounding factors).

A receiver operating characteristics (ROC) analysis was performed to determine a cut-off value for daily moderate-to-vigorous physical activity. Thus, three subgroups could be identified according to activity level (low PA defined as MVPA less than 30 min/day based on the recommendation of the WHO [18], moderate PA defined as MVPA from 30 to 54 min daily and high PA defined as MVPA over 54 min, both based on the cut-off value of the ROC analysis.

We used the Kruskal–Wallis test to see whether the degree of depression varied significantly at these different levels of physical activity. To adjust the *α*-level when using multiple tests, we applied the Bonferroni correction [41].

Cohen’s *d*-analysis was used to calculate the effect sizes [42] of the difference in outcome variables between the groups with different activity levels. To investigate the hypothesis that the relationship between depression and MVPA is mediated by self-esteem, a mediator analysis was performed. The mediation models were calculated using the Haye’s PROCESS v3 macro for SPSS [43]. The 95% confidence interval of the indirect effect was estimated using bootstrapping based on 1000 iterations.

## Results

**Fig. 1:**
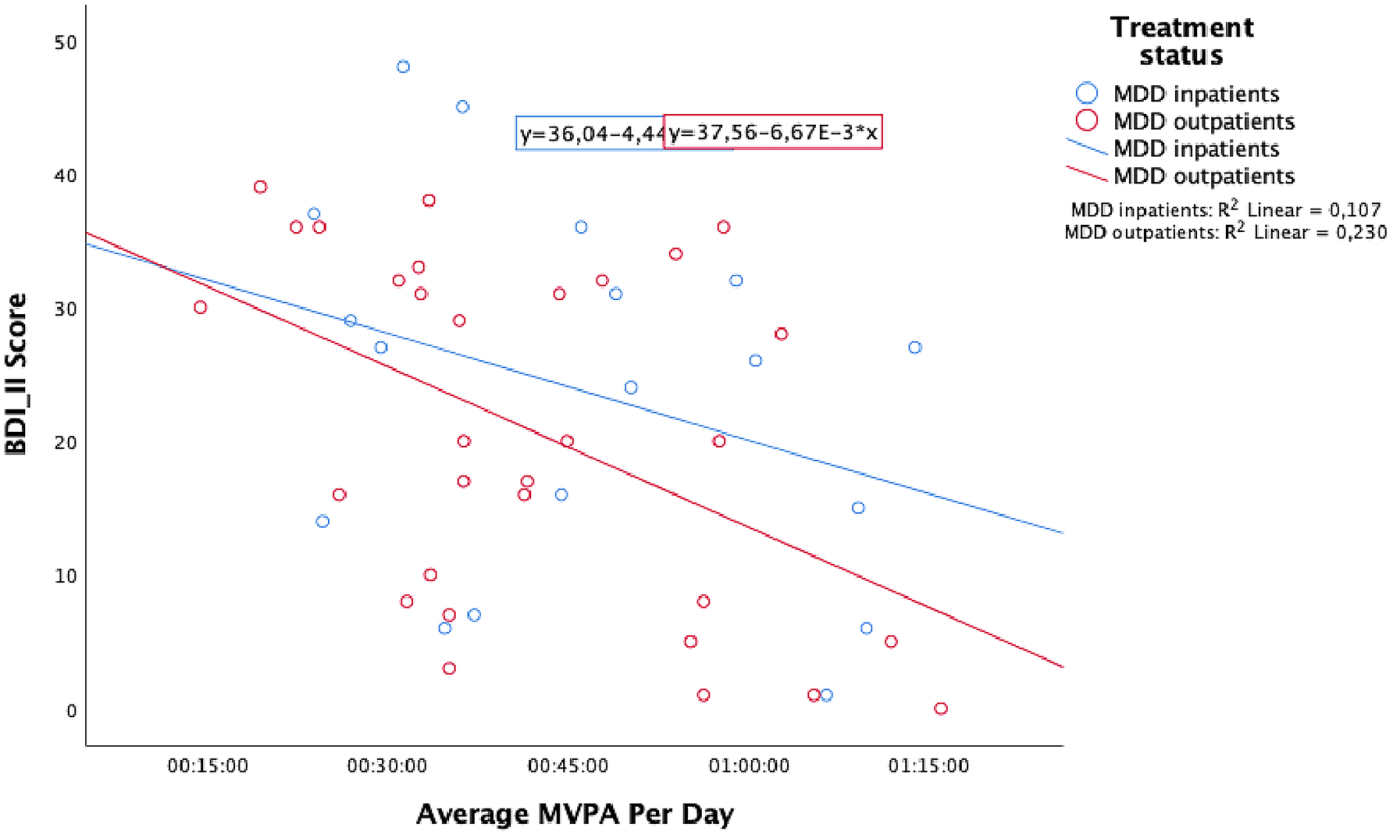
Regression analysis between depression score (BDI-II) and average MVPA per day for in- and outpatients

A linear regression was calculated to predict the BDI score based on MVPA and a significant regression equation was found (*R*^2^ = 0.23, *F*(1,29) = 8.66, *p* < 0.01) (see Fig. 1).

The results of the multivariable linear regression model are presented in Table 2. MVPA and age were significant predictors of the BDI-II score (*R*^2^ = 0.32, *F*(3,45) = 7.07, *p* < 0.001).

**Table 2:** Regression model of the predictors of the BDI-II score

Predictor	Estimate	*p* value
Constant	64,525	< 0.001
Average MVPA per day	− 0.006	< 0.001
Gender	− 5.363	0.169
Age	− 0.410	< 0.01

MVPA was measured in hours, minutes and seconds. Gender was coded 1 for females and 2 for males. The age was stated in years

A Mann–Whitney *U* test was calculated to determine if there were differences in the BDI-II scores between in- and outpatients. There was no statistically significant difference in the BDI-II score (*U* = 604.00, *Z* = − 0.62, *p* = 0.54) and in the MVPA (as measured by accelerometers) between both groups (*U* = 242.00, *Z* = − 0.93, *p* = 0.35).

In contrast to this, the aerobic capacity, as measured by *V*O_2max_ ml/(min kg), differed statistically significant for in- and outpatients (*U* = 22.00, *Z* = − 2.39, *p* = 0.02).

We did neither observe a significant correlation in the inpatients group between MVPA and BDI-II (*r* = − 0.34, *p* = 0.18), nor between MVPA and *V*O_2max_ ml/(min kg) (*r* = − 0.09, *p* = 0.87) or BDI-II and *V*O_2max_ ml/(min kg) (*r* = − 0.60, *p* = 0.21) (see Table 3).

**Table 3:** Spearman correlation of moderate-to-vigorous physical activity and selected characteristics among inpatients with major depressive disorder

	MVPA	BDI II	*V*O_2max_ ml/(min kg)	MSWS GSW
MVPA		− 0.34^(*N*=18)^	− 0.60^(*N*=6)^	0.21^(*N*=17)^
BDI II			− 0.09^(*N*=6)^	− 0.66**^(*N*=27)^
*V*O_2max_ ml/(min kg)				0.71^(*N*=6)^

*p* < 0.05* *p* < 0.01** *p* < 0.001*** sample sizes vary as not all patients participated in all investigations completely

In the outpatients’ group, there was a negative correlation between *V*O_2max_ ml/(min kg) and BDI-II (*r* = − 0.49, *p* = 0.04) and between MVPA and BDI-II (*r* = − 0.47, *p* < 0.01). There was no correlation between MVPA and *V*O_2max_ ml/(min kg), *r* = 0.26, *p* = 0.38 (see Table 4).

**Table 4:** Spearman correlation of moderate-to-vigorous physical activity and selected characteristics among outpatients with major depressive disorder

	MVPA	BDI II	*V*O_2max_ ml/(min kg)	MSWS GSW
MVPA		− 0.47**^(*N*=31)^	0.26^(N=14)^	0.38*^(*N*=30)^
BDI II			− 0.49*^(*N*=17)^	− 0.65**^(*N*=42)^
*V*O_2max_ ml/(min kg)				0.37^(*N*=17)^

*p* < 0.05* *p* < 0.01** *p* < 0.001*** sample sizes vary as not all patients participated in all investigations completely

Taking into account the possible confounding factor of medication (CPZ equivalent) not correlating with the BDI-II (*r* = 0.20, *p* = 0.20) or MVPA (*r* = − 0.26, *p* = 0.14), the relationship between MVPA and BDI was confirmed [partial correlation (*r* = − 0.47, *p* < 0.01)]. The same applies to the AMI equivalent, which showed no correlation with the BDI-II (*r* = − 0.14, *p* = 0.35) or MVPA (*r* = − 0.05, *p* = 0.77) and thus appears to have no influence on the relationship between MVPA and BDI-II [partial correlation (*r* = − 0.53, *p* < 0.01)].

**Fig. 2:**
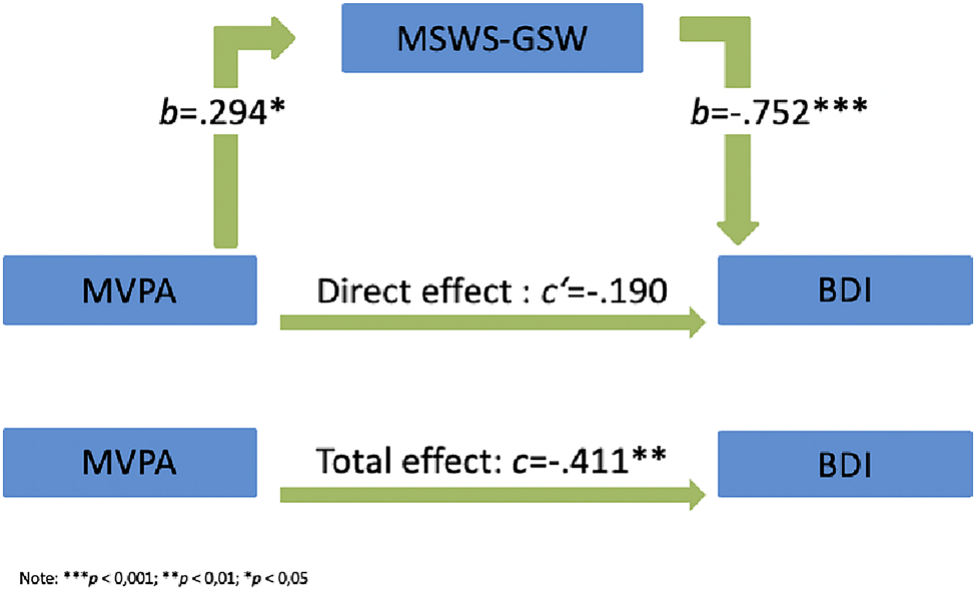
Mediation analysis: self-esteem effecting the correlation between MVPA and BDI-II

A mediation analysis was performed to investigate whether MVPA predicts depression symptoms and whether the direct path is mediated by self-esteem (see Fig. 2). We observed an effect of MVPA on the BDI-II, *c* = − 0.41, *p* < 0.01. After entering the mediator into the model, MVPA predicted the mediator “self-esteem” significantly *b* = 0.29, *p* < 0.05, which in turn predicted BDI-II score significantly, *b* = − 0.75, *p* < 0.001. We found that the relationship between MVPA and the BDI-II score is fully mediated by self-esteem, indirect effect *ab* = − 0.22, 95% confidence interval *b* = − 0.41 to *b* = − 0.04.

**Fig. 3:**
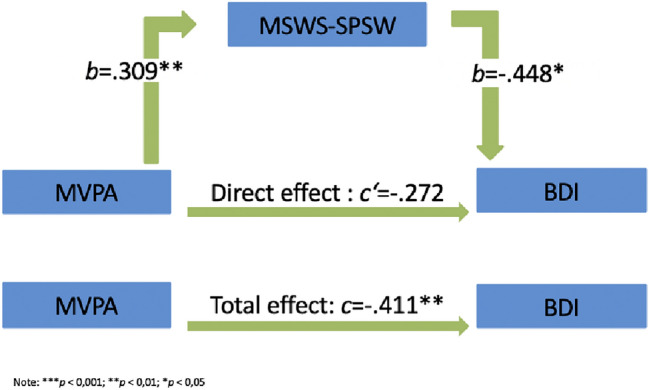
Mediation analysis: physical self-perception effecting the correlation between MVPA and BDI-II

This mediation could be shown more accurately when physical self-perception was taken into account as a mediator (see Fig. 3). The relationship between MVPA and the BDI-II score is also fully mediated by physical self-perception, indirect effect *ab* = − 0.14, 95%-confidence interval *b* = − 0.34 to *b* = − 0.01.

**Fig. 4:**
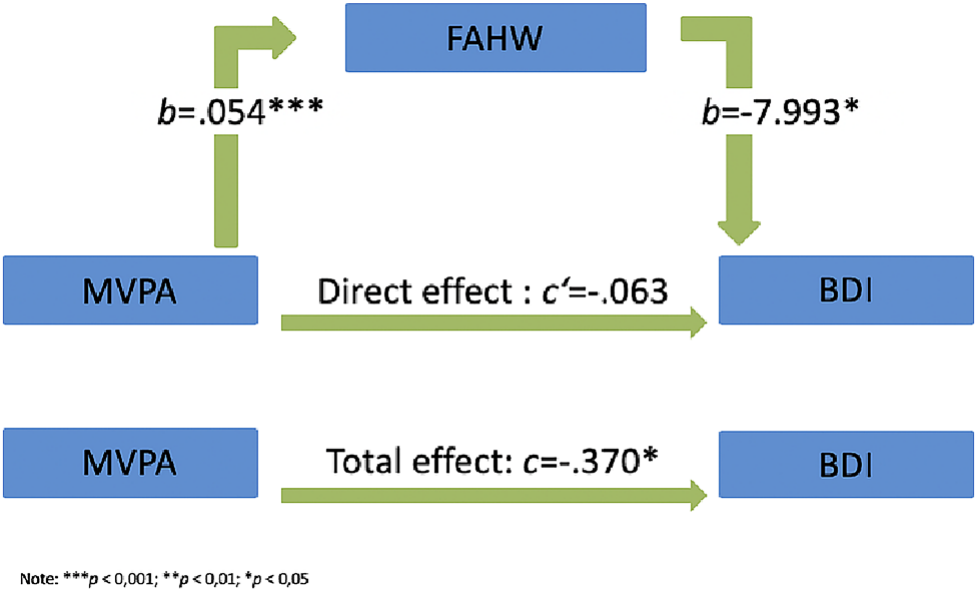
Mediation analysis: general well-being effecting the correlation between MVPA and BDI-II

Figure 4 shows the results for the mediator effect of general well-being on the relationship between MVPA and the BDI-II score. General well-being proved to be the total mediator of the relationship between MVPA and the BDI-II score. The unstandardized regression coefficient between MVPA and the BDI-II score decreases from *c* = − 0.37 to *c*′ = − 0.06, when general well-being was taken into account as a mediator. This reduction turns out to be significant, indirect effect: *ab* = − 0.43, 95% confidence interval: *b* = − 0.67 to *b* = − 0.14.

The results of the mediator analysis are shown in Figs. 2 and 3.

To better elucidate the relationship between daily moderate-to-vigorous physical activity and symptom severity, we divided all patients into two groups according to BDI-II scores. The cut-off for this was set at a BDI-II score of 13 points, defining the threshold between minimal depression (*n* = 19, *M* = 5.16, SD = 3.11) and mild depression (*n* = 55, *M* = 28.11, SD = 8.15). Subsequent ROC (receiver operating characteristics) analysis revealed a cut-off value of 54.31 min of daily moderate-to-vigorous physical activity.

**Fig. 5:**
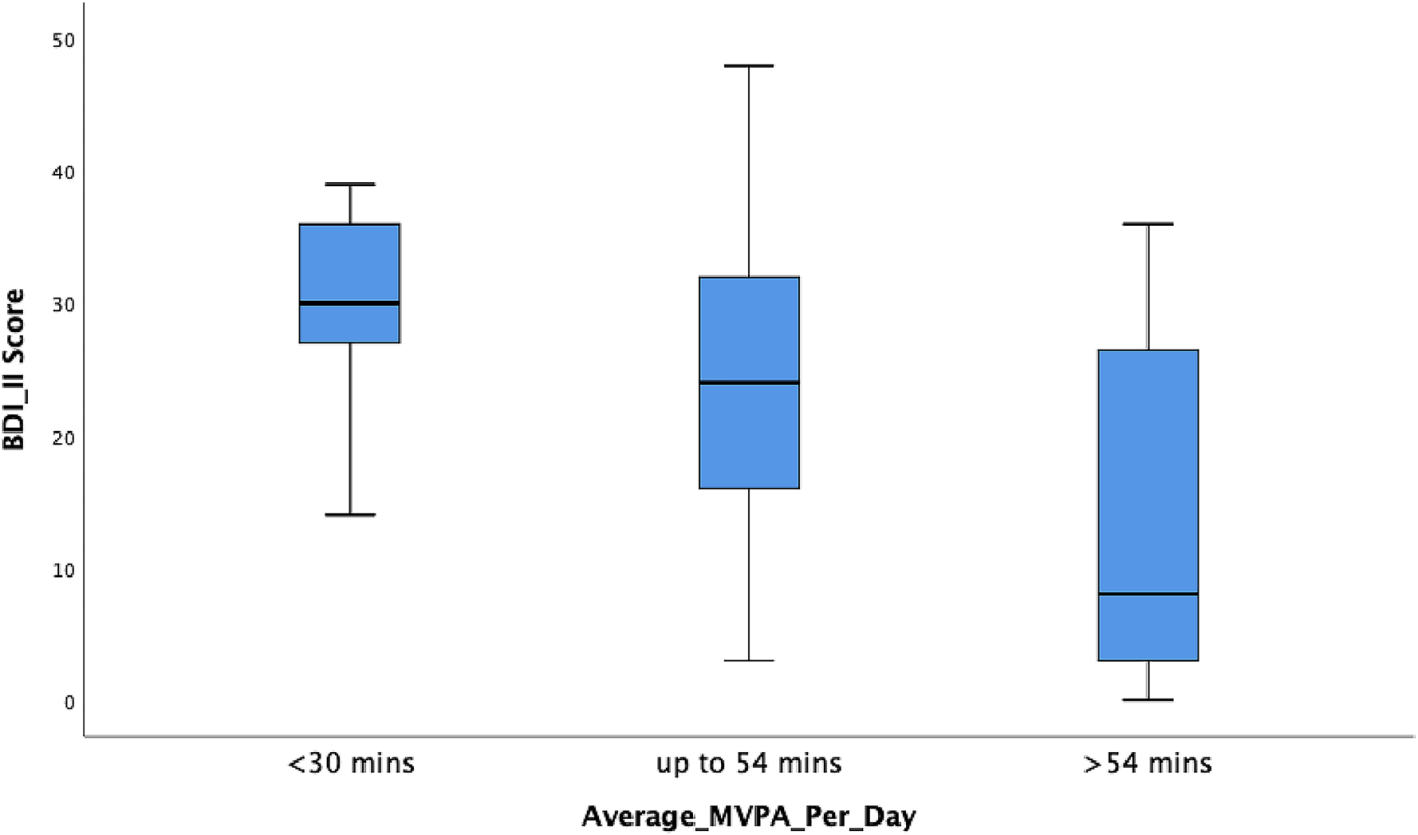
Boxplots with whiskers: differences in the level of depression for different levels of physical activity

Physical activity was divided into one of three categories: low activity defined as MVPA less than 30 min/day (*n* = 9, *M* = 29.33, SD = 9.08) based on the recommendations of the WHO, moderate activity defined as MVPA up to 54 min daily (*n* = 25, *M* = 23.64, SD = 12.73) and high activity defined as MVPA over 54 min daily (*n* = 15, *M* = 14.07, SD = 12.87) based on our calculated cut-off value. A Kruskal–Wallis test confirmed that the level of depression differed statistically significant for the different levels of physical activity (chi-square = 9.19, *p* = 0.010, illustrated in Fig. 5). Subsequent post hoc tests (Dunn–Bonferroni tests) showed that the group with moderate and the group with high activity differ marginally significant (*z* = 2.33, *p* = 0.05) as well as the group with low and the group with high activity (*z* = 2.82, *p* = 0.01), effect intensity according to Cohen: *r* = 0.350 and *r* = 0.565). A possible confounding effect of medication (CPZ and AMI equivalents) on the group comparison of depression severity for different levels of physical activity was controlled using a Kruskal–Wallis test and no differences were found between the activity groups in relation to medication (Kruskal–Wallis-*H* = 4.56, *p* = 0.10 for CPZ equivalent and Kruskal–Wallis-*H* = 0.75, *p* = 0.69 for AMI equivalent).

## Discussion

The current study is, to our knowledge, the first to compare the impact of physical activity on self-esteem, physical self-perception, general well-being and depression symptoms between inpatients and outpatients with MDD.

### Effects of moderate-to-vigorous PA on depressive symptoms

As hypothesized, we observed a significant negative correlation between moderate-to-vigorous physical activity and depression severity score (BDI-II) in outpatients. Interestingly, we did not observe this correlation for the inpatient group even though there was no statistically significant difference in MVPA between both groups.

One explanation for this observation is the psychosocial aspect of PA, meaning social ties and integration into social networks, which are not provided equally in the inpatient and outpatient setting. This is underlined by the mediating influence of well-being and self-esteem which partially explains the influence of MVPA in our study cohort of outpatients.

Kawachi et al. [44] developed a so-called main effect model, which describes how participation in social networks can directly generate positive affective states, such as increased self-esteem, which in turn can benefit mental health due to a proactive lifestyle (e.g., regular exercise) and the modulation of the neuroendocrine response to stress. Moreover, studies have already linked positive perceptions of the social environment (i.e., emotional and social support) with higher levels of physical activity and a lower risk of cardiovascular disease [45, 46]. Regarding the lastingness of physical activity engagement, studies have also shown that high levels of emotional support from friends and family lead to the recommended physical activities being maintained during follow-up examinations [47, 48]. Hallgren et al. [49] investigated in an RCT with 946 adults with mild-to-moderate depression longitudinal associations between the availability of social networks and the severity of depression after a 12-week intervention (cognitive behavioural therapy and exercise). The results of the study revealed that participants with greater access to supportive social relationships reported greater exercise-induced improvements in depression than participants with “low” availability of relationships. Similarly, a cross-sectional study involving 40,000 subjects showed that a higher level of social support and social engagement partially mediated the association between leisure activity and depression, while biological models such as increased parasympathetic vagal tone (resting heart rate) or changes in metabolism had virtually no effect on this association [50]. Likewise, in our study, cardiorespiratory fitness was not associated with subjective well-being. Despite showing no differences in the sociodemographic characteristic, this might potentially explain the correlations observed in outpatients, assuming a denser and more accessible social network and the ability to participate in combined social and physical activities.

Regarding potential implementation in daily clinical practice, this could mean that the therapeutic outcomes of physical activity and exercise in an inpatient setting could be increased by establishing positive relationships with significant others and integration in a social network.

Even though CRF was not associated with subjective well-being, there was still a correlation with the BDI-II score. Thus, while physical activity appears to have a short-term effect on depression symptoms in modifying subjective well-being, fitness may have a longer term effect independent of current well-being.

### Effects of self-esteem and general habitual well-being on the relationship between depressive symptoms and MVPA

As an explanatory approach for the observed significant negative correlation between moderate-to-high levels of physical activity and the severity of depression (BDI II), we used factors such as self-esteem and general habitual well-being in a mediation analysis to investigate how subjects in outpatient settings benefit from physical activity.

The mediator analysis identified the variable self-esteem as a total mediator of the relationship between MVPA and depression symptoms. Even more profoundly, we found out that physical self-perception also seems to be a total mediator, which speaks for an important role of physical activity related positive physical perceptions and body satisfaction.

Leading in the same direction, a longitudinal study with 126 healthy controls already showed that the relationship between movement time and affect is mediated by physical self-perception (physical attractiveness, sporting competence) [51]. Further data from the National Longitudinal Study of Adolescent Health show that family cohesion, parent–child communication and parental engagement positively predict MVPA and that self-esteem mediates the relationship between parental influence and physical activity [52].

In another step, we explored the mediating effect of general well-being as another contributory variable on the association between MVPA and depression symptoms. General well-being was also strongly connected to physical activity and proved to be a total mediator of the relationship between MVPA and depression symptoms. Our data thus extend earlier findings from non-patient populations by connecting activity induced well-being with the severity of depression symptoms. Studies on healthy subjects were so far only able to show that the values of MVPA were higher in participants with subjective well-being [53, 54].

Taken together, recent evidence suggests that PA interventions should therefore focus more on subjective outcomes like the perception of well-being and self-esteem rather than analyzing solely objective data for physical fitness or changes in BMI to increase the effects of physical activity on depressive symptoms. In conjunction with this, we were also able to show, as mentioned earlier, that higher *V*O_2max_ values and thus a better CRF are associated with lower BDI-II scores, but this does not seem to have an immediate but longer term effect on subjective well-being.

### Effects of activity levels on depression symptoms

Finally, we examined whether the recommendations of the WHO regarding physical activity also apply to patients with MDD and whether achieving these recommendations is sufficient to reduce depressive symptoms. Overall, our analysis shows an inverse association between the severity of depression and the levels of physical activity in both, in- and outpatients. More precisely, we first determined that a threshold from none-to-mild depression (displayed in a BDI-II score of 13 points) is exceeded from 54.31 min of daily moderate-to-vigorous physical activity. Based on this cut-off, weekly MVPA levels exceeding more than twice the volume of current recommendations would be sufficient to influence depression symptoms. A further analysis (not shown) showed that the level of depression varied significantly between non-adherers to current recommendations, with less than 30 min of daily activity, recommendation adherers, with 30–54 min, and subjects overreaching current recommendations with more than 54 min daily.

These results are in line with the study by Hallgren et al. [55] who investigated the association between regular physical activity and the onset of depression. In a cohort study of 43,863 Swedish adults over 13 years, they found that those who exceeded the recommended weekly duration (≥ 300 min/week) had a 29% reduced risk of depression. In a meta-analysis of prospective studies, Schuch and colleagues [56] also investigated associations between physical activity and depression, including sub-analyses by duration of activity (> 150 min versus less) and reported that higher physical activity was associated with a significantly lower probability of developing depression. However, these analyses are primarily based on unspecific definitions of activity levels (‘high’ or ‘low’) without precise specification of the duration in minutes and the applied intensity.

Altogether, the correlation between physical activity and depression was also confirmed in our study, but with a special focus on the depressive symptoms of patients with already medically diagnosed MDD. Furthermore, we succeeded in defining the activity level for the depiction of depression more precisely in terms of a BDI-II score of over 13 points.

### Strengths and limitations

Previous studies have tended to use self-report methods such as physical activity questionnaires, which are characterized by their poor reliability and validity [23]. Measuring physical activity through the use of accelerometers and recording physical performance using spiroergometry are, therefore, key strengths of this study, as they are objective methods that overcome the limitations of self-reporting and represent valid and reliable recordings [57, 58].

Another point that has to be mentioned are the significantly different *V*O_2max_ and thus the physical fitness between in- and outpatients. This might be an explanation for the lack of influence of physical fitness in our study populations. Interestingly, data from a large population-based prospective cohort study [59] showed that the association between cardiorespiratory fitness and incident depressive symptoms is independent of MVPA. Eventually, the identification of the main psycho-biological pathways involved remains to be clarified in further studies.

As this study is based on cross-sectional data and has some methodological limitations, the results should be interpreted with caution. The sample is small and is locally limited to the Department of Psychiatry at the University Hospital in Frankfurt am Main. For this reason, the findings presented cannot be generalized. Especially in the measurement of aerobic capacity, it is important to emphasize that the number of patients who underwent this examination was very limited to 24 participants. In particular, the associations tested based on these data should be interpreted with utmost caution. Nevertheless, we did not want to withhold these data and further research should confirm the associations with testing a larger number of samples.

Moreover, it is not possible for us to draw firm conclusions about the direction of possible causalities in any of the associations described, it is possible and likely that the actual relationship between acute depression and physical activity should be considered bidirectional. For example, the question remains unanswered whether PA causes improved depression symptoms or whether improved depression symptoms are associated with increased PA inversely. Consequently, longitudinal studies or RCT designs are necessary to prove our preliminary data and conclusions.

## Conclusion

We examined the relationship between moderate-to-vigorous physical activity and depression symptoms and critically reviewed the recommendations for physical activity for healthy populations in patients with MDD. The key findings of the study were (1) that in the sample studied, patients in outpatient settings benefit more from MVPA regarding their depression symptoms than inpatients and (2) that self-esteem, physical self-awareness and general habitual well-being play a major role as mediators of this relationship. Additionally, we were able to show that there is an inverse relationship between the severity of depression and the different levels of physical activity in both inpatients and outpatients. We, therefore, suggest that patients with depression symptoms should participate in physical activity with moderate-to-vigorous intensity and durations of at least 54 min daily.

These positive results are encouraging and suggest that further studies should investigate potential sources that might lead to the differential findings.

Taken together, intervention strategies that promote MVPA levels exceeding the duration recommended for general health (≥ 30 min/day) with a special focus on self-esteem may reduce depression symptoms in patients with MDD.
